# G-Protein-Coupled Lysophosphatidic Acid Receptors and Their Regulation of AKT Signaling

**DOI:** 10.3390/ijms17020215

**Published:** 2016-02-05

**Authors:** Anjum Riaz, Ying Huang, Staffan Johansson

**Affiliations:** 1 Institute of Biochemistry and Biotechnology, University of the Punjab, 54590 Lahore, Pakistan; anjum.ibb@pu.edu.pk; 2 Department of Medical Biochemistry and Microbiology, Uppsala University, Biomedical Center, 75123 Uppsala, Sweden; ying.huang@imbim.uu.se

**Keywords:** G-protein coupled receptors (GPCR), lysophosphatidic acid (LPA), PI3K, AKT

## Abstract

A hallmark of G-protein-coupled receptors (GPCRs) is their ability to recognize and respond to chemically diverse ligands. Lysophospholipids constitute a relatively recent addition to these ligands and carry out their biological functions by activating G-proteins coupled to a large family of cell-surface receptors. This review aims to highlight salient features of cell signaling by one class of these receptors, known as lysophosphatidic acid (LPA) receptors, in the context of phosphatidylinositol 3-kinase (PI3K)–AKT pathway activation. LPA moieties efficiently activate AKT phosphorylation and activation in a multitude of cell types. The interplay between LPA, its receptors, the associated Gαi/o subunits, PI3K and AKT contributes to the regulation of cell survival, migration, proliferation and confers chemotherapy-resistance in certain cancers. However, detailed information on the regulation of PI3K–AKT signals induced by LPA receptors is missing from the literature. Here, some urgent issues for investigation are highlighted.

## 1. Introduction

**Figure 1:**
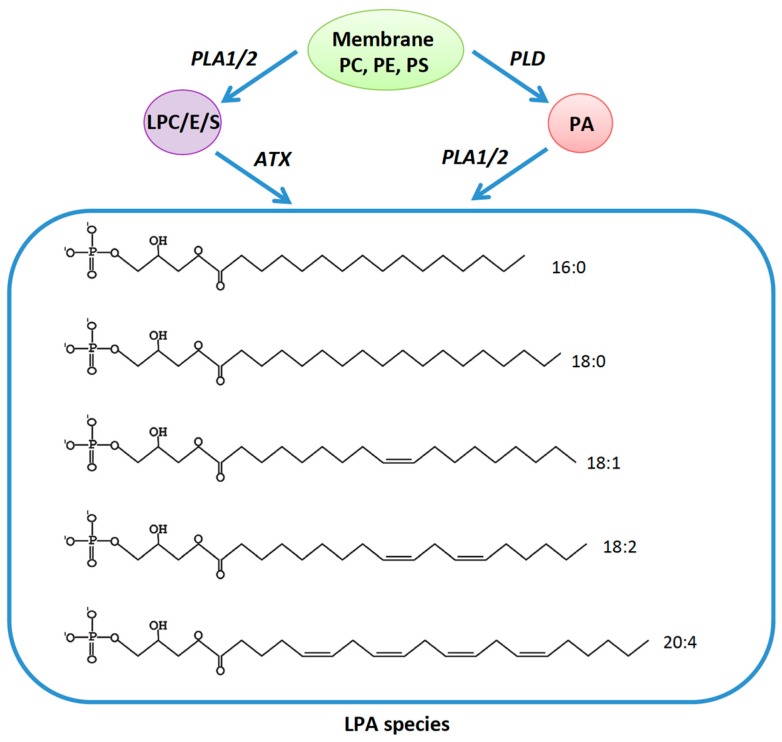
Biosynthesis of lysophosphatidic acid (LPA): The membrane phospholipids phosphatidyl choline (PC), phosphatidyl ethanolamine (PE) or phosphatidyl serine (PS) get converted into their corresponding lyso-forms by the action of phospholipase A1 and A2 (PLA1/2). Autotaxin (ATX) then generates different LPA species using the lyso-forms of membrane lipids. Alternatively, PC, PE or PS are catalyzed into phosphatidic acid (PA) by phospholipase D (PLD). PLA1/2 then acts on phosphatidic acid (PA) and form LPA. The structure of some common human LPAs is shown.

Lipid signaling through G-protein-coupled receptors has relatively recently added a dimension to signaling research. The focus of this review is a diverse set of G-protein-coupled receptors (GPCRs) that respond to the glycerophospholipid lysophosphatidic acid (LPA) and activate the phosphatidylinositol 3-kinase (PI3K)–AKT signaling pathway among others. LPA may potentially have a large impact on this important pathway, and thereby on cancer and several other diseases. LPA is widely present in almost all types of mammalian tissues examined [1,2]. The highest concentration of LPA is present in serum (10 µM) and it has also been found in other body fluids and tissues including blood plasma, saliva, cerebrospinal fluid and semen [3,4,5,6]. LPA is not a single molecule but a group of small sized species (molecular weight: 430–480 Da) having a glycerol backbone substituted with a phosphate group and an acyl chain in position 1 or 2 [7]. In humans the most abundant species of LPA is the 16:0 form, *i.e.* it contains a palmityl chain [1]. However, the term LPA is usually used to refer to the 18:1 species, which is also commonly used as a research reagent [5]. Other reported LPA moieties are 18:0, 18:2, 16:1 and 20:4 [7] (Figure 1).

All LPA species are biosynthesized via two major metabolic routes (Figure 1). Depending upon the site of synthesis, membrane phospholipids get converted to the corresponding lysophospholipids by the action of phospholipase A1 (PLA1), phospholipase A2 (PLA2), or PLA1 and lecithin-cholesterol acyltransferase (LCAT). Autotaxin (ATX) then acts on the lysophospholipids and converts them into LPA species [8,9]. The second pathway first converts the phospholipids into phosphatidic acid by the action of phospholipase D. Then PLA1 or PLA2 metabolize phosphatidic acid to the lysophosphatidic acids [3]. Extensive discussion of LPA metabolism is not a subject of this review but has been elegantly detailed elsewhere [1,10].

The ATX-associated LPA production and signaling has gained tremendous importance in recent studies [11,12]. Initial data identified ATX as a factor responsible for enhanced cellular motility of melanoma cells. This activity was connected to G protein signaling based on the finding that pertussis toxin abrogated ATX-induced cell migration [13]. Further research revealed the expression pattern of ATX and established it as a major metabolic regulator of LPA generation [9,14,15,16]. ATX-generated LPA activity is vital for maintenance of proper vascular and neural development [4,10,17,18,19,20,21,22]. It is also implicated in the regulation of inflammatory responses including lymphocyte adhesion, as well as in proper bone development and reproduction, *etc*. [23,24,25,26]. In the pathophysiology of cancer, the ATX–LPA axis is considered important for cell proliferation, migration and invasion in a multitude of cancer types including breast, colon, lung and liver [27,28,29,30,31,32]. ATX activity and LPA levels are up-regulated in response to VEGF stimulation [33]. Furthermore, LPA up-regulates mRNA and protein levels of VEGF, which constitutes a vascular homeostasis-maintaining circuit [34].

Some recent studies have implicated LPA in emergence of chemotherapy drug resistance in cancer cells [35,36]. Venkatraman and colleagues used established breast cancer cell lines and a mouse model to show that LPA protects breast tumors from the destructive effects of doxorubicin by enhancing the expression and activity of NRF2-regulated antioxidant gene products. They also reported up-regulation of transporters widely recognized as major contributors to multidrug resistance in cancer cells [37]. Others have proposed a role for LPA in chemotherapy resistance of other types of cancers [36].

**Figure 2:**
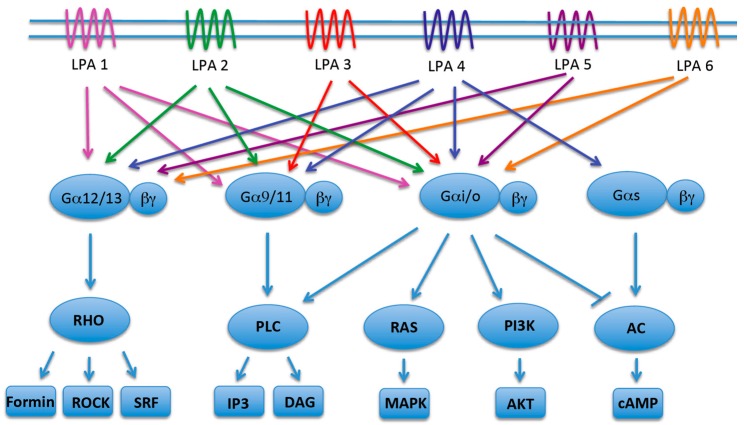
Cell surface LPA receptors and their downstream signaling pathways: LPA signaling is mediated by six known 7-TM receptors that are coupled to members from at least two of four trimeric G-protein families. LPA signaling regulates critical cellular responses such as cytoskeletal changes, cell motility, proliferation, and resistance to apoptosis.

The biological functions of LPA are mediated by at least six recognized cell-surface receptors [5]. The genes encoding these proteins are designated as *LPAR1-6* in humans and *Lpar1-6* in mice, while the receptors are termed as LPA1-6 [38]. All LPA receptors are rhodopsin-like 7-TM proteins that signal through at least two of the four Gα subunit families (Gα12/13, Gαq/11, Gαi/o and GαS) [1,5]. LPA receptors usually trigger response from multiple heterotrimeric G-proteins, resulting in diverse outcomes in a context and cell type dependent manner (Figure 2) [39].

Gα12/13-mediated LPA signaling regulates cell migration, invasion and cytoskeletal re-adjustments through activation of RHO pathway proteins [40]. RAC activation downstream of Gαi/o–PI3K also regulates similar processes, but the most notable function of LPA-induced Gαi/o is mitogenic signaling through the RAF–MEK–MAPK cascade and survival signaling through the PI3K–AKT pathway [41,42,43,44]. The LPA-coupled Gαq/11 protein primarily regulates Ca^2+^ homeostasis through PLC and the second messengers IP3 and DAG [45,46,47]. Lastly, GαS can activate adenylyl cyclase and increase cAMP concentration upon LPA stimulation [48]. However, the same enzyme is also inhibited by Gαi/o, underscoring the complexity of signaling activity triggered downstream LPA receptors [49]. This aspect in terms of LPA-induced PI3K–AKT activation will be discussed in more details in a later section.

## 2. LPA Receptors 1-3 (The EDG Family)

LPA1-3 are also referred as the endothelial differentiation, G-protein-coupled (EDG) family of LPA receptors. This is because these were first identified as orphan GPCRs involved in endothelial gene differentiation in human umbilical vein endothelial cells [50]. Evidence that LPA is the ligand for one of these receptors (EDG2) came from the work of Jerold Chun and co-workers, who in 1996 showed that the previously known ventricular zone gene-1 encodes a functional G-protein-coupled LPA receptor in neuroblast cells [51]. This was followed by the identification of EDG1 as a functional sphingosine-1-phosphate (S1P) receptor [52]. Two more EDG-type receptors were subsequently identified as LPA2 (EDG4) and LPA3 (EDG7) based on homology to LPA1 [53,54].

LPA1 is a 41-kD protein that is widely expressed, albeit at different levels, in all human adult tissues examined [55]. The importance of LPA1 signaling during development and adult life has been demonstrated through numerous approaches. In developing mouse fetus, *Lpar1* is highly expressed in the nervous system [20,38]. In knockout experiments, 50% *Lpar1*^−/−^ mice exhibited perinatal lethality and those that survived displayed retarded growth compared to the wild-type mice and other development abnormalities [56].

LPA1 carries out downstream signaling by the activation of members from three of the four Gα subunit families (Gα 12/13, Gαq/11 and Gαi/o) [39]. LPA1 has not been shown to activate GαS. The pathways generated downstream of LPA1 regulate cytoskeletal organization and cell migration, cell proliferation, apoptosis, cell survival and Ca^2+^ homeostasis [5,57,58]. LPA1 also lowers cAMP levels by inhibiting adenylyl cyclase, thus indirectly affecting a range of cAMP-regulated cellular processes [49]. Recently, crystal structures of antagonist-bound LPA1 were provided by Hanson and colleagues that will facilitate deeper understanding of the structural basis of LPA1 functions. According to their model, LPA1 exhibits similar as well as dissimilar features compared to the related S1P receptor. The extracellular ligand-binding pocket of LPA1 appears more flexible, enabling LPA1 to bind more diverse chemical moieties including ligands that can bind to the related cannabinoid receptor CB1 [59].

LPA2 in humans is a 39-kD protein and consists of 351 amino acids. This receptor shares ~55% amino acid sequence homology with LPA1 [5]. In adult humans, high level expression of LPA2 has been reported in leukocytes and testis. Moderate level expression is found in prostate, spleen, thymus and pancreas [55]. LPA2 is not expressed by cells of other vital organs such as brain, heart, *etc.* In mice, high level expression of *Lpar2* observed at embryonic stage rapidly decreases at birth, and except for mouse kidney, testis and uterus, other adult tissues examined exhibit moderate to low levels of *Lpar2* expression [60]. *Lpar2*^−/−^ mice are viable and healthy, while those null for both *Lpar1* and *Lpar2* present with features essentially consistent with those of *Lpar1*^−/−^ [61]. These data indicate functional redundancy of LPA2 with LPA1. In terms of signaling activity, LPA2 mostly activates the same pathways as triggered by LPA1 [5,39]. However, a notable difference is the unique cross-talk behavior attributed to LPA2. In this context, LPA2 regulates cell migration through interaction with thyroid receptor-interacting protein 6 (TRIP6), promoted by phosphorylation of TRIP6 by SRC [62,63]. Interestingly, while LPA1 was reported to promote motility of pancreatic cancer cells, LPA2 activation inhibited EGF-induced migration and invasion of these cells downstream the Gα12/13-RHO cascade [32,64]. In contrast, LPA2 promotes LPA-stimulated migration of gastric cancer cells [29]. This raises the possibility of finding further diversity in biological effects of LPA signaling in cancers and other diseases.

Human LPA3 is a 40-kD protein comprising 353 amino acids and shares sequence homology with LPA1 (~54%) and LPA2 (~49%) [5]. In adult humans *LPAR3* is highly expressed in heart, pancreas, prostate and testis. Moderate levels of expression are also found in brain, lungs and ovary [55]. In other tissues its presence is either negligible or absent. During mouse development high level *Lpar3* expression has been observed in embryonic heart and kidney. After birth the expression becomes more widely distributed and high to moderate *Lpar3* levels are found in most adult mouse tissues [5,60].

*Lpar3*^−/−^ mice are viable and normal, but the female null mice exhibit reproductive system abnormalities [56]. Evidence from such mouse studies and LPA level estimations in healthy human females and pregnant women has highlighted the importance of this lipid in the maintenance of proper female reproductive physiology [25].

The signaling activity of LPA3 results from its coupling to Gαi/o and Gαq/11. Through these Gα proteins LPA3 can trigger the activation of PLC and MAPK. Its activity also regulates Ca^+2^ homeostasis and cAMP levels in the cells [65].

## 3. LPA Receptors 4-6 (The Non-EDG Family)

Clues pointing towards the existence of unknown LPA receptors came from work on rodent cells that did not express the classical LPA receptors and LPA1/LPA2 double-null fibroblasts lacking LPA3 expression, both of which gave excellent response to LPA stimulation [66,67]. Furthermore, platelets responded to a chemically distinct alkylated form of LPA not preferred by LPA1-3, indicating presence of another LPA-accepting moiety [68].

The first so-called non-EDG LPA receptor was identified by Kyoko Noguchi and his colleagues in 2003. Named as LPA4, the receptor shares little homology (~20%) with the EDG family of LPA receptors. However, LPA4 showed similarity to the previously known P2Y family of purinergic receptors [69]. In humans LPA4 has a molecular weight of 42-kD and comprises 370 amino acids and the gene encoding this receptor is highly expressed in ovaries [5,70]. Moderate to low level expression has also been found in other tissues. The mouse gene appears more widely expressed during embryonic stage and in adult tissues [71]. Increased prenatal death has been noted in *Lpar4^−/−^* mice compared to normal, but the majority of them survives and is normal [66,71]. In terms of signaling function LPA4 has been reported to associate with all four Gα subunit families, triggering a multitude of cascades in different cells [72]. Most notably, LPA4 is the only receptor that activates adenylyl cyclase and thus causes a rise in cAMP levels in cells [48]. It also enhances cell-adhesion and is known to negatively regulate cell migration [73].

LPA5 is one of the two more recently identified LPA receptors [70]. This protein comprising 372 amino acids shares around ~35% homology with LPA4 [5]. *Lpar5* is widely expressed and distributed in adult and developing mice. It is also highly expressed in different parts of developing brain [38,60]. In humans high *LPAR5* expression is limited to spleen. *Lpar5^−/−^* mice are viable and normal and do not exhibit any obvious phenotype after birth [56]. The signaling activity of LPA5 is mediated by Gα12/13 and Gαq/11 [67]. It has been implicated in the regulation of water absorption, Ca^2+^ mobilization and increase in cAMP levels [70]. Activation of LPA5 inhibits matrix metalloproteases and negatively affects motility of 3T3 and rat sarcoma cells [74,75]. In a recent study, this receptor was shown to block migration and matrigel invasion of melanoma cells. *Lpar5^−/−^* null mice also exhibit reduced lung metastasis by melanoma cells compared to wild type cells [76]. These data merit further detailed investigations into the role of LPA5 in human cancers and other diseases.

**Table 1:** Expression pattern of LPA receptors and their known physiological functions in mice and humans.

Receptor	Species	Major Expression Sites	Biological Functions	References
LPA1	Mouse	Brain, heart, lungs, stomach, kidneys, spleen, uterus, testes	Neurodevelopment regulation; neural cell proliferation, differentiation and migration; astrocyte proliferation	[1,5,20,38,55,56]
	Human	Brain, heart, lungs, stomach, intestine, placenta, kidneys, spleen, uterus, testis		
LPA2	Mouse	Kidney, uterus, brain, testes	Cell survival; cell migration; immune system regulation	[1,5,23,43,55,56]
	Human	Leukocytes, spleen, thymus, pancreas, brain, prostate, testes		
LPA3	Mouse	Lungs, kidney, uterus, small intestine, testes	Male and female reproductive system regulation; embryo implantation	[1,5,25,55,56]
	Human	Heart, testes, prostate, pancreas, brain		
LPA4	Mouse	Heart, skin, ovary, thymus, lungs, kidney	Blood and lymphatic vessel development; neurite retraction; cell adhesion	[17,60,66,67,71]
	Human	Ovary, thymus, brain, heart, testes, prostate, spleen		
LPA5	Mouse	Heart, lung, stomach, small intestine, liver, spleen, platelets, mast cells	Neurite retraction; inhibition of cell migration; calcium level regulation; water absorption; platelet activation; mast cell activation	[60,66,67,70]
	Human	Heart, small intestine, colon, liver, spleen		
LPA6	Mouse	Hair, skin	Hair development	[66,70,77,78]
	Human	Hair, immune cells		

LPA6 is the most recently reported LPA receptor, which was first identified as an LPA-binding P2Y5 family protein vital for hair growth and quickly confirmed as a RHO-activating LPA receptor by Satoshi Ishii and colleagues [77,78]. So far, researchers have been able to couple LPA6 with RHO signaling through Gα12/13 [79]. This implies that future studies will uncover further details of LPA6 biology and functions. The tissue distribution and main function of the different LPA receptors are summarized in Table 1.

## 4. PI3K-AKT Pathway and Its Regulation

The PI3K-AKT pathway generates signals regulating a wide range of reactions, in particular events involved in cell survival and metabolism. AKT activation is regulated at several levels, and defect regulation of the PI3K-AKT pathway is linked to diseases including cancer, diabetes, and atherosclerosis [80,81,82]. Elevated AKT activity is commonly seen in metastatic tumors and continues to be a topic under intense research since AKT makes important contributions both to the invasive behavior of the cells and to their resistance to anti-tumor medical treatment [83].

The available information on the regulation of the PI3K-AKT pathway is becoming increasingly complex. Actually, the concept of one pathway may be considered obsolete since the different isoforms of PI3K and AKT are regulated differently and mediate different functions. Early on, some of the key steps were identified, which can be summarized in the following points. Some PI3Ks can be activated by binding of the regulatory subunit to specific phosphorylated tyrosines in cell surface receptors or adaptor proteins and by binding of the catalytic subunit to GTP-RAS [84,85]. The generation of PI3,4P_2_ and PI3,4,5P_3_ by PI3K creates binding sites for AKT at the plasma membrane and enables the subsequent activating phosphorylation of AKT at Thr308 in the kinase domain by PDK1 and Ser473 in the so called hydrophobic motif, mainly by TORC2 [86,87]. PTEN negatively regulates the pathway by dephosphorylating the inositol 3’-phosphate group [88]. Together, this information formed a basic model for AKT activation.

However, the mechanism for PI3K-AKT activation exhibits stimuli-specific variations. The different PI3Ks are activated in receptor-specific manners and by distinct GTPases of the RAS and RHO families. Some well-documented examples are the selective activation of enzymes containing the catalytic subunit p110α or p110δ by tyrosine kinase receptors, while p110β and p110γ PI3Ks are activated by GPCRs [44]. Integrins also induce PI3K activity, which at least for β1-integrins occurs through p110α [89,90]. p110α and p110γ can interact with several RAS proteins, including H-, N-, K-, R-RAS and TC21, while p110δ interacts with R-RAS and TC21 [91]. In contrast, p110β does not interact with RAS proteins, but is instead regulated by RAC and CDC42 [92].

The three AKT isoforms have been reported to localize to the cytoplasm, the plasma membrane, the nucleus and mitochondria with different preferences [93]. They appear to elicit partly overlapping responses, but the regulation of epithelial-mesenchymal transition (EMT) and E-cadherin expression are examples where AKT1 and 2 can induce opposite responses [94]. Although the PI3Ks discussed above catalyze the same reaction, 3’-phosphorylation of phosphatidylinositols, which is essential for the phosphorylation of AKT at Thr308 and Ser473, the AKT regulatory steps downstream of PI3K have been described to vary depending on the initial receptor stimuli. PAK and Freud1/Aki1 were shown to have scaffolding functions necessary for PDGF- and EGF-induced AKT activation, respectively, a role also ascribed to β-arrestin-2 during insulin stimulation [95,96,97]. Furthermore, multiple additional modifications of AKTs besides phosphorylation of Thr308 and Ser473 have been reported, including tyrosine phosphorylations, ubiquitinations, SUMOylation, and O-GlcNAcylation [98]. Among these, K63-linked ubiquitination has been shown to be critically required for AKT phosphorylation and activation upon certain stimuli, *i.e.*, LPS, IL-1, EGF, and IGF-1 [98]. Three phosphatases (PP2A, PHLPP 1, and PHLPP 2) and a de-ubiquitinase (CYLD) have been identified to act on AKT, adding further diversity to the regulation of AKT activity [84,98,99]. Whether the various posttranslational modifications are specific for particular AKT isoforms remain to be established.

## 5. The LPA-PI3K-AKT Signaling Axis

LPA stimulation results in robust signaling from PI3K and resultant AKT phosphorylation on Thr308 and Ser473 [90,100]. As described above, this pathway is coupled to the LPA receptors through the Gαi/oβγ proteins, and notably, Gαi/oβγ can be activated by all six LPA receptors. The PI3K catalytic isoform that is activated by a majority of GPCRs, including LPA receptors, is p110β [44]. LPA-activated AKT primarily contributes to cell survival but may also provide inputs towards other processes such as cell migration and proliferation [43,101]. In Schwann cells, AKT activation promotes cell survival and also affects differentiation in response to LPA stimulation [57,102]. Recently, it was shown that LPA protects cervical cancer cells from Cisplatin-mediated cell death through a PI3K-AKT pathway [36]. Interestingly, Murga and colleagues have shown that LPA-induced AKT activation is mediated by the Gβγ complex and not the Gα subunit [103]. The exact mechanistic details of how this is regulated remain obscure.

**Figure 3:**
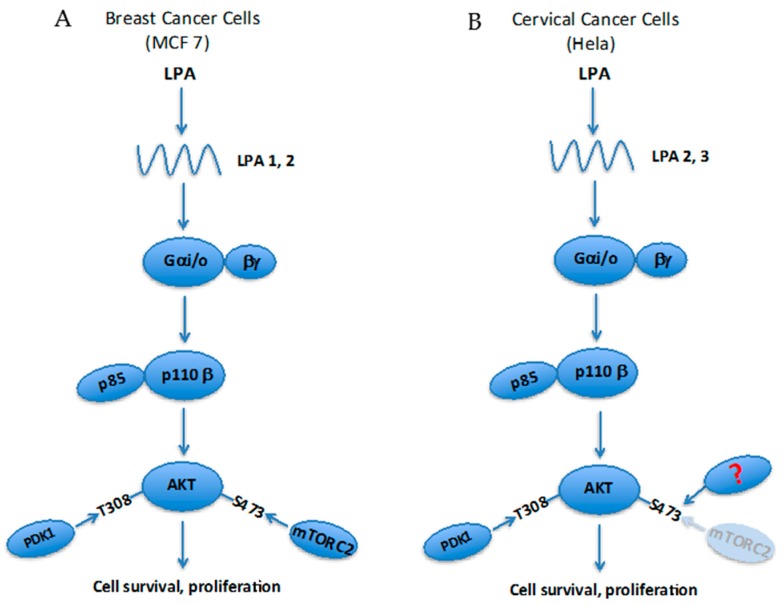
Cell-type dependent regulation of AKT S473 phosphorylation by LPA: (**A**) Knockdown of RICTOR using a specific siRNA disrupts kinase activity of mTORC2. In MCF7 cells (breast cancer cells) this abrogates phosphorylation on Ser473 located in the AKT hydrophobic motif; (**B**) The same approach has no affect on AKT Ser473 phosphorylation in Hela cells (cervical cancer cells). This indicates the presence of an alternate pathway(s) and reflects different LPA receptor expression pattern in these cells; This pathway utilizes another, yet unidentified, kinase (marked with ?) for the Ser473 phosphorylation than mTORC2.

LPA signaling through Gαi/oβγ also results in MAPK pathway activation, and there is some evidence of cross-talk with PI3K–AKT [42]. P38MAPK has been shown as vital for LPA- or S1P-induced AKT Ser473 phosphorylation in different cancer cell lines, where P38MAPK was proposed as the kinase responsible for this hydrophobic motif modification [104]. This is in contrast to the now widely accepted view that mTORC2 is responsible for Ser473 phosphorylation on AKT in mammalian and insect cells [86]. However, as recently shown by us, this regulatory phosphorylation is complex and other kinases than mTORC2 may be preferred by cells under different situations [90]. In terms of LPA signaling, we found that AKT phospho-Ser473 levels remain unaffected after mTORC2 inactivation by RICTOR knockdown in HeLa cells while it is significantly reduced in MCF7 cells. These results possibly reflect diverging signaling pathways triggered by distinct LPA receptors expressed by these cells (Figure 3). This situation should be considered when anti-tumor treatments are designed [105]. In fact, it has been reported that LPA receptors are highly expressed in drug-resistant cervical cancer cells and confer this resistance through AKT [36]. This supports the hypothesis that the type of LPA receptor expressed by cells is a major determinant of LPA-PI3K–AKT survival signaling.

## 6. Future Prospects

The importance of LPA-induced AKT functions has not received proper attention until relatively recently, and several key questions need to be clarified.

(1) Which AKT isoform(s) is/are activated by each of the different LPA receptors? It is becoming increasingly clear that AKT isoforms perform overlapping as well as isoform-specific functions in cells. Elucidating and understanding these functions is vital due to the involvement of PI3K–AKT pathway in a number of diseases.

(2) The importance of ubiquitination for AKT phosphorylation after several stimuli has recently been described. Is ubiquitination involved in the AKT regulation downstream LPA receptors as well? If so, by which ubiquitin ligase(s)?

(3) Are the regulatory mechanisms for AKT activation used by the six LPA receptors, the same or different in any respect? This issue is important to clarify, in order to efficiently target the LPAR–PI3K–AKT axis during tumor treatment.

(4) A more detailed understanding of cross-talk between LPA receptors and other cell-surface receptors is imperative for broader understanding of cellular signaling from this class of GPCRs.

## Abbreviations

GPCRs: G-protein-coupled receptors
LPA: Lysophosphatidic acid
PI3K: Phosphatidyl inositol 3-kinase
ATX: Autotaxin
EDG: Endothelial differentiation genes
TORC2: Target of rapamycin complex 2

## References

[1] Choi J.W., Herr D.R., Noguchi K., Yung Y.C., Lee C.W., Mutoh T., Lin M.E., Teo S.T., Park K.E., Mosley A.N. (2010). Lpa receptors: Subtypes and biological actions. Annu. Rev. Pharmacol. Toxicol..

[2] Moolenaar W.H. (1999). Bioactive lysophospholipids and their G protein-coupled receptors. Exp. Cell Res..

[3] Aoki J., Taira A., Takanezawa Y., Kishi Y., Hama K., Kishimoto T., Mizuno K., Saku K., Taguchi R., Arai H. (2002). Serum lysophosphatidic acid is produced through diverse phospholipase pathways. J. Biol. Chem..

[4] Choi J.W., Chun J. (2013). Lysophospholipids and their receptors in the central nervous system. Biochim. Biophys. Acta.

[5] Yung Y.C., Stoddard N.C., Chun J. (2014). Lpa receptor signaling: Pharmacology, physiology, and pathophysiology. J. Lipid Res..

[6] Hama K., Bandoh K., Kakehi Y., Aoki J., Arai H. (2002). Lysophosphatidic acid (Lpa) receptors are activated differentially by biological fluids: Possible role of lpa-binding proteins in activation of lpa receptors. FEBS Lett..

[7] Bandoh K., Aoki J., Taira A., Tsujimoto M., Arai H., Inoue K. (2000). Lysophosphatidic acid (Lpa) receptors of the edg family are differentially activated by lpa species: Structure–activity relationship of cloned Lpa receptors. FEBS Lett..

[8] Aoki J., Inoue A., Okudaira S. (2008). Two pathways for lysophosphatidic acid production. Biochim. Biophys. Acta (BBA).

[9] Moolenaar W.H., Perrakis A. (2011). Insights into autotaxin: How to produce and present a lipid mediator. Nat. Rev..

[10] Blaho V.A., Hla T. (2011). Regulation of mammalian physiology, development, and disease by the sphingosine 1-phosphate and lysophosphatidic acid receptors. Chem. Rev..

[11] Okudaira S., Yukiura H., Aoki J. (2010). Biological roles of lysophosphatidic acid signaling through its production by autotaxin. Biochimie.

[12] Houben A.J., Moolenaar W.H. (2011). Autotaxin and lpa receptor signaling in cancer. Cancer Metastasis Rev..

[13] Stracke M.L., Krutzsch H.C., Unsworth E.J., Arestad A., Cioce V., Schiffmann E., Liotta L.A. (1992). Identification, purification, and partial sequence analysis of autotaxin, a novel motility-stimulating protein. J. Biol. Chem..

[14] Lee H.Y., Murata J., Clair T., Polymeropoulos M.H., Torres R., Manrow R.E., Liotta L.A., Stracke M.L. (1996). Cloning, chromosomal localization, and tissue expression of autotaxin from human teratocarcinoma cells. Biochem. Biophys. Res. Commun..

[15] Perrakis A., Moolenaar W.H. (2014). Autotaxin: Structure-function and signaling. J. Lipid Res..

[16] Zhang Y., Chen Y.C., Krummel M.F., Rosen S.D. (2012). Autotaxin through lysophosphatidic acid stimulates polarization, motility, and transendothelial migration of naive t cells. J. Immunol..

[17] Teo S.T., Yung Y.C., Herr D.R., Chun J. (2009). Lysophosphatidic acid (lpa) in vascular development and disease. IUBMB Life.

[18] Fukushima N., Ye X., Chun J. (2002). Neurobiology of lysophosphatidic acid signaling. The Neurosci.: Rev. J. Bringing Neurobiol. Neurol. Psychiatry.

[19] Spohr T.C., Choi J.W., Gardell S.E., Herr D.R., Rehen S.K., Gomes F.C., Chun J. (2008). Lysophosphatidic acid receptor-dependent secondary effects via astrocytes promote neuronal differentiation. J. Biol. Chem..

[20] Ye X., Fukushima N., Kingsbury M.A., Chun J. (2002). Lysophosphatidic acid in neural signaling. Neuroreport.

[21] Yung Y.C., Mutoh T., Lin M.E., Noguchi K., Rivera R.R., Choi J.W., Kingsbury M.A., Chun J. (2011). Lysophosphatidic acid signaling may initiate fetal hydrocephalus. Sci. Transl. Med..

[22] Yung Y.C., Stoddard N.C., Mirendil H., Chun J. (2015). Lysophosphatidic acid signaling in the nervous system. Neuron.

[23] Knowlden S., Georas S.N. (2014). The autotaxin-lpa axis emerges as a novel regulator of lymphocyte homing and inflammation. J. Immunol..

[24] Gennero I., Laurencin-Dalicieux S., Conte-Auriol F., Briand-Mesange F., Laurencin D., Rue J., Beton N., Malet N., Mus M., Tokumura A. (2011). Absence of the lysophosphatidic acid receptor lpa1 results in abnormal bone development and decreased bone mass. Bone.

[25] Ye X., Chun J. (2010). Lysophosphatidic acid (Lpa) signaling in vertebrate reproduction. Trends Endocrinol. Metab.: TEM.

[26] Sims S.M., Panupinthu N., Lapierre D.M., Pereverzev A., Dixon S.J. (2013). Lysophosphatidic acid: A potential mediator of osteoblast-osteoclast signaling in bone. Biochim. Biophys. Acta.

[27] Tsujiuchi T., Araki M., Hirane M., Dong Y., Fukushima N. (2014). Lysophosphatidic acid receptors in cancer pathobiology. Histol. Histopathol..

[28] Tsujiuchi T., Hirane M., Dong Y., Fukushima N. (2014). Diverse effects of lpa receptors on cell motile activities of cancer cells. J. Recept. Signal Transduct. Res..

[29] Yang D., Yang W., Zhang Q., Hu Y., Bao L., Damirin A. (2013). Migration of gastric cancer cells in response to lysophosphatidic acid is mediated by lpa receptor 2. Oncol. Lett..

[30] Sokolov E., Eheim A.L., Ahrens W.A., Walling T.L., Swet J.H., McMillan M.T., Simo K.A., Thompson K.J., Sindram D., McKillop I.H. (2013). Lysophosphatidic acid receptor expression and function in human hepatocellular carcinoma. J. Surg. Res..

[31] Popnikolov N.K., Dalwadi B.H., Thomas J.D., Johannes G.J., Imagawa W.T. (2012). Association of autotaxin and lysophosphatidic acid receptor 3 with aggressiveness of human breast carcinoma. Tumour Biol.: J. Int. Soc. Oncodev. Biol. Med..

[32] Komachi M., Sato K., Tobo M., Mogi C., Yamada T., Ohta H., Tomura H., Kimura T., Im D.S., Yanagida K. (2012). Orally active lysophosphatidic acid receptor antagonist attenuates pancreatic cancer invasion and metastasis *in vivo*. Cancer Sci..

[33] Sako A., Kitayama J., Shida D., Suzuki R., Sakai T., Ohta H., Nagawa H. (2006). Lysophosphatidic acid (Lpa)-induced vascular endothelial growth factor (Vegf) by mesothelial cells and quantification of host-derived vegf in malignant ascites. J. Surg. Res..

[34] Lin C.I., Chen C.N., Huang M.T., Lee S.J., Lin C.H., Chang C.C., Lee H. (2008). Lysophosphatidic acid up-regulates vascular endothelial growth factor-c and lymphatic marker expressions in human endothelial cells. Cell. Mol. Life Sci..

[35] Samadi N., Bekele R., Capatos D., Venkatraman G., Sariahmetoglu M., Brindley D.N. (2011). Regulation of lysophosphatidate signaling by autotaxin and lipid phosphate phosphatases with respect to tumor progression, angiogenesis, metastasis and chemo-resistance. Biochimie.

[36] Sui Y., Yang Y., Wang J., Li Y., Ma H., Cai H., Liu X., Zhang Y., Wang S., Li Z. (2015). Lysophosphatidic acid inhibits apoptosis induced by cisplatin in cervical cancer cells. BioMed Res. Int..

[37] Venkatraman G., Benesch M.G., Tang X., Dewald J., McMullen T.P., Brindley D.N. (2015). Lysophosphatidate signaling stabilizes nrf2 and increases the expression of genes involved in drug resistance and oxidative stress responses: Implications for cancer treatment. FASEB J. Off. Publ. Federation Am. Soc. Exp. Biol..

[38] Sheng X., Yung Y.C., Chen A., Chun J. (2015). Lysophosphatidic acid signalling in development. Development.

[39] Fukushima N., Ishii S., Tsujiuchi T., Kagawa N., Katoh K. (2015). Comparative analyses of lysophosphatidic acid receptor-mediated signaling. Cell. Mol. Life Sci. CMLS.

[40] Xiang S.Y., Dusaban S.S., Brown J.H. (2013). Lysophospholipid receptor activation of rhoa and lipid signaling pathways. Biochim. Biophys. Acta.

[41] van Leeuwen F.N., Olivo C., Grivell S., Giepmans B.N.G., Collard J.G., Moolenaar W.H. (2003). Rac activation by lysophosphatidic acid lpa1receptors through the guanine nucleotide exchange factor tiam1. J. Biol. Chem..

[42] Fang X., Yu S., LaPushin R., Lu Y., Furui T., Penn L.Z., Stokoe D., Erickson J.R., Bast R.C., Mills G.B. (2000). Lysophosphatidic acid prevents apoptosis in fibroblasts via g(I)-protein-mediated activation of mitogen-activated protein kinase. Biochem. J..

[43] Ye X., Ishii I., Kingsbury M.A., Chun J. (2002). Lysophosphatidic acid as a novel cell survival/apoptotic factor. Biochim. Biophys. Acta.

[44] Guillermet-Guibert J., Bjorklof K., Salpekar A., Gonella C., Ramadani F., Bilancio A., Meek S., Smith A.J., Okkenhaug K., Vanhaesebroeck B. (2008). The p110beta isoform of phosphoinositide 3-kinase signals downstream of g protein-coupled receptors and is functionally redundant with p110gamma. Proc. Natl. Acad. Sci. USA.

[45] Hildebrandt J.P. (1995). Lysophosphatidic acid induces inositol phosphate and calcium signals in exocrine cells from the avian nasal salt gland. J. Membarin Biol..

[46] Gennero I., Xuereb J.-M., Simon M.-F., Girolami J.-P., Bascands J.-L., Chap H., Boneu B., Sié P. (1999). Effects of lysophosphatidic acid on proliferation and cytosolic ca^++^ of human adult vascular smooth muscle cells in culture. Thromb. Res..

[47] Litosch I. (2009). Phosphatidic acid potentiates gαq stimulation of phospholipase c-β1 signaling. Biochem. Biophys. Res. Commun..

[48] Lee C.-W., Rivera R., Dubin A.E., Chun J. (2007). Lpa4/gpr23 is a lysophosphatidic acid (Lpa) receptor utilizing gs-, gq/gi-mediated calcium signaling and g12/13-mediated rho activation. J. Biol. Chem..

[49] Wittpoth C., Scholich K., Yigzaw Y., Stringfield T.M., Patel T.B. (1999). Regions on adenylyl cyclase that are necessary for inhibition of activity by βγ and g(Iα) subunits of heterotrimeric g proteins. Proc. Natl. Acad. Sci. USA.

[50] Takuwa Y., Takuwa N., Sugimoto N. (2002). The edg family g protein-coupled receptors for lysophospholipids: Their signaling properties and biological activities. J. Biochem..

[51] Hecht J.H., Weiner J.A., Post S.R., Chun J. (1996). Ventricular zone gene-1 (vzg-1) encodes a lysophosphatidic acid receptor expressed in neurogenic regions of the developing cerebral cortex. J. Cell Biol..

[52] Lee M.-J., Van Brocklyn J.R., Thangada S., Liu C.H., Hand A.R., Menzeleev R., Spiegel S., Hla T. (1998). Sphingosine-1-phosphate as a ligand for the g protein-coupled receptor edg-1. Science.

[53] Contos J.J.A., Chun J. (2000). Genomic characterization of the lysophosphatidic acid receptor gene, lpa2/edg4, and identification of a frameshift mutation in a previously characterized cdna. Genomics.

[54] Bandoh K., Aoki J., Hosono H., Kobayashi S., Kobayashi T., Murakami-Murofushi K., Tsujimoto M., Arai H., Inoue K. (1999). Molecular cloning and characterization of a novel human g-protein-coupled receptor, edg7, for lysophosphatidic acid. J. Biol. Chem..

[55] Archbold J.K., Martin J.L., Sweet M.J. (2014). Towards selective lysophospholipid gpcr modulators. Trends Pharmacol. Sci..

[56] Choi J.W., Lee C.W., Chun J. (2008). Biological roles of lysophospholipid receptors revealed by genetic null mice: An update. Biochim. Biophys. Acta.

[57] Anliker B., Choi J.W., Lin M.E., Gardell S.E., Rivera R.R., Kennedy G., Chun J. (2013). Lysophosphatidic acid (lpa) and its receptor, lpa1, influence embryonic schwann cell migration, myelination, and cell-to-axon segregation. Glia.

[58] Sakai N., Chun J., Duffield J.S., Wada T., Luster A.D., Tager A.M. (2013). Lpa1-induced cytoskeleton reorganization drives fibrosis through ctgf-dependent fibroblast proliferation. FASEB J. Off. Publ. Federation Am. Soc. Exp. Biol..

[59] Chrencik J.E., Roth C.B., Terakado M., Kurata H., Omi R., Kihara Y., Warshaviak D., Nakade S., Asmar-Rovira G., Mileni M. (2015). Crystal structure of antagonist bound human lysophosphatidic acid receptor 1. Cell.

[60] Ohuchi H., Hamada A., Matsuda H., Takagi A., Tanaka M., Aoki J., Arai H., Noji S. (2008). Expression patterns of the lysophospholipid receptor genes during mouse early development. Dev. Dyn..

[61] Contos J.J., Ishii I., Fukushima N., Kingsbury M.A., Ye X., Kawamura S., Brown J.H., Chun J. (2002). Characterization of Lpa(2) (Edg4) and Lpa(1)/Lpa(2) (Edg2/Edg4) lysophosphatidic acid receptor knockout mice: Signaling deficits without obvious phenotypic abnormality attributable to Lpa(2). Mol. Cell. Biol..

[62] Xu J., Lai Y.J., Lin W.C., Lin F.T. (2004). Trip6 enhances lysophosphatidic acid-induced cell migration by interacting with the lysophosphatidic acid 2 receptor. J. Biol. Chem..

[63] Lai Y.J., Chen C.S., Lin W.C., Lin F.T. (2005). C-src-mediated phosphorylation of trip6 regulates its function in lysophosphatidic acid-induced cell migration. Mol. Cell. Biol..

[64] Komachi M., Tomura H., Malchinkhuu E., Tobo M., Mogi C., Yamada T., Kimura T., Kuwabara A., Ohta H., Im D.S. (2009). Lpa1 receptors mediate stimulation, whereas lpa2 receptors mediate inhibition, of migration of pancreatic cancer cells in response to lysophosphatidic acid and malignant ascites. Carcinogenesis.

[65] Hama K., Aoki J. (2010). Lpa3, a unique g protein-coupled receptor for lysophosphatidic acid. Prog. Lipid Res..

[66] Yanagida K., Ishii S. (2011). Non-edg family lpa receptors: The cutting edge of lpa research. J. Biochem..

[67] Ishii S., Noguchi K., Yanagida K. (2009). Non-edg family lysophosphatidic acid (lpa) receptors. Prostaglandins Lipid Mediat..

[68] Pamuklar Z., Lee J.S., Cheng H.-Y., Panchatcharam M., Steinhubl S., Morris A.J., Charnigo R., Smyth S.S. (2008). Individual heterogeneity in platelet response to lysophosphatidic acid: Evidence for a novel inhibitory pathway. Arterioscler. Thromb. Vasc. Biol..

[69] Noguchi K., Ishii S., Shimizu T. (2003). Identification of p2y9/gpr23 as a novel g protein-coupled receptor for lysophosphatidic acid, structurally distant from the edg family. J. Biol. Chem..

[70] Yanagida K., Kurikawa Y., Shimizu T., Ishii S. (2013). Current progress in non-edg family lpa receptor research. Biochim. Biophys. Acta.

[71] Sumida H., Noguchi K., Kihara Y., Abe M., Yanagida K., Hamano F., Sato S., Tamaki K., Morishita Y., Kano M.R. (2010). Lpa4 regulates blood and lymphatic vessel formation during mouse embryogenesis. Blood.

[72] Yanagida K., Ishii S., Hamano F., Noguchi K., Shimizu T. (2007). Lpa4/p2y9/gpr23 mediates rho-dependent morphological changes in a rat neuronal cell line. J. Biol. Chem..

[73] Lee Z., Cheng C.-T., Zhang H., Subler M.A., Wu J., Mukherjee A., Windle J.J., Chen C.-K., Fang X. (2008). Role of lpa(4)/p2y9/gpr23 in negative regulation of cell motility. Mol. Biol. Cell.

[74] Yanagida K., Masago K., Nakanishi H., Kihara Y., Hamano F., Tajima Y., Taguchi R., Shimizu T., Ishii S. (2009). Identification and characterization of a novel lysophosphatidic acid receptor, p2y5/lpa6. J. Biol. Chem..

[75] Pasternack S.M., von Kugelgen I., Al Aboud K., Lee Y.A., Ruschendorf F., Voss K., Hillmer A.M., Molderings G.J., Franz T., Ramirez A. (2008). G protein-coupled receptor p2y5 and its ligand lpa are involved in maintenance of human hair growth. Nat. Genet..

[76] Araki M., Kitayoshi M., Dong Y., Hirane M., Ozaki S., Mori S., Fukushima N., Honoki K., Tsujiuchi T. (2014). Inhibitory effects of lysophosphatidic acid receptor-5 on cellular functions of sarcoma cells. Growth Fact..

[77] Dong Y., Hirane M., Araki M., Fukushima N., Tsujiuchi T. (2014). Lysophosphatidic acid receptor-5 negatively regulates cellular responses in mouse fibroblast 3t3 cells. Biochem. Biophys. Res. Commun..

[78] Lee S.C., Fujiwara Y., Liu J., Yue J., Shimizu Y., Norman D.D., Wang Y., Tsukahara R., Szabo E., Patil R. (2015). Autotaxin and lpa1 and lpa5 receptors exert disparate functions in tumor cells versus the host tissue microenvironment in melanoma invasion and metastasis. Mol. Cancer Res. MCR.

[79] Lee M., Choi S., Hallden G., Yo S.J., Schichnes D., Aponte G.W. (2009). P2y5 is a g(alpha)i, g(alpha)12/13 g protein-coupled receptor activated by lysophosphatidic acid that reduces intestinal cell adhesion. Am. J. Physiol. Gastrointest. Liver Physiol..

[80] Gao J., Aksoy B.A., Dogrusoz U., Dresdner G., Gross B., Sumer S.O., Sun Y., Jacobsen A., Sinha R., Larsson E. (2013). Integrative analysis of complex cancer genomics and clinical profiles using the cbioportal. Sci. Signal..

[81] Schultze S.M., Jensen J., Hemmings B.A., Tschopp O., Niessen M. (2011). Promiscuous affairs of pkb/akt isoforms in metabolism. Arch. Physiol. Biochem..

[82] Yu H., Littlewood T., Bennett M. (2015). Akt isoforms in vascular disease. Vasc. Pharmacol..

[83] Dibble C.C., Manning B.D. (2009). A molecular link between akt regulation and chemotherapeutic response. Cancer Cell.

[84] Newton A.C., Trotman L.C. (2014). Turning off akt: Phlpp as a drug target. Annu. Rev. Pharmacol. Toxicol..

[85] Vanhaesebroeck B., Guillermet-Guibert J., Graupera M., Bilanges B. (2010). The emerging mechanisms of isoform-specific pi3k signalling. Nat. Rev. Mol. Cell Biol..

[86] Sarbassov D.D., Guertin D.A., Ali S.M., Sabatini D.M. (2005). Phosphorylation and regulation of akt/pkb by the rictor-mtor complex. Science.

[87] Jacinto E., Facchinetti V., Liu D., Soto N., Wei S., Jung S.Y., Huang Q., Qin J., Su B. (2006). Sin1/mip1 maintains rictor-mtor complex integrity and regulates akt phosphorylation and substrate specificity. Cell.

[88] Song M.S., Salmena L., Pandolfi P.P. (2012). The functions and regulation of the pten tumour suppressor. Nat. Rev. Mol. Cell Biol..

[89] Zeller K.S., Idevall-Hagren O., Stefansson A., Velling T., Jackson S.P., Downward J., Tengholm A., Johansson S. (2010). Pi3-kinase p110α mediates β1 integrin-induced akt activation and membrane protrusion during cell attachment and initial spreading. Cell Signal..

[90] Riaz A., Zeller K.S., Johansson S. (2012). Receptor-specific mechanisms regulate phosphorylation of akt at ser473: Role of rictor in β1 integrin-mediated cell survival. PLoS ONE.

[91] Rodriguez-Viciana P., Sabatier C., McCormick F. (2004). Signaling specificity by ras family gtpases is determined by the full spectrum of effectors they regulate. Mol. Cell. Biol..

[92] Fritsch R., de Krijger I., Fritsch K., George R., Reason B., Kumar M.S., Diefenbacher M., Stamp G., Downward J. (2013). Ras and rho families of gtpases directly regulate distinct phosphoinositide 3-kinase isoforms. Cell.

[93] Santi S.A., Lee H. (2010). The akt isoforms are present at distinct subcellular locations. Am. J. Physiol..

[94] Villagrasa P., Diaz V.M., Vinas-Castells R., Peiro S., Del Valle-Perez B., Dave N., Rodriguez-Asiain A., Casal J.I., Lizcano J.M., Dunach M. (2012). Akt2 interacts with snail1 in the e-cadherin promoter. Oncogene.

[95] Higuchi M., Onishi K., Kikuchi C., Gotoh Y. (2008). Scaffolding function of pak in the pdk1-akt pathway. Nat. Cell Biol..

[96] Nakamura A., Naito M., Tsuruo T., Fujita N. (2008). Freud-1/aki1, a novel pdk1-interacting protein, functions as a scaffold to activate the pdk1/akt pathway in epidermal growth factor signaling. Mol. Cell. Biol..

[97] Luan B., Zhao J., Wu H., Duan B., Shu G., Wang X., Li D., Jia W., Kang J., Pei G. (2009). Deficiency of a β-arrestin-2 signal complex contributes to insulin resistance. Nature.

[98] Chan C.-H., Jo U., Kohrman A., Rezaeian A.H., Chou P.-C., Logothetis C., Lin H.-K. (2014). Posttranslational regulation of akt in human cancer. Cell Biosci..

[99] Lim J.H., Jono H., Komatsu K., Woo C.-H., Lee J., Miyata M., Matsuno T., Xu X., Huang Y., Zhang W. (2012). Cyld negatively regulates transforming growth factor-β-signalling via deubiquitinating akt. Nat. Commun..

[100] Kang Y.C., Kim K.M., Lee K.S., Namkoong S., Lee S.J., Han J.A., Jeoung D., Ha K.S., Kwon Y.G., Kim Y.M. (2004). Serum bioactive lysophospholipids prevent trail-induced apoptosis via pi3k/akt-dependent cflip expression and bad phosphorylation. Cell Death Differ..

[101] Kim E.K., Yun S.J., Do K.H., Kim M.S., Cho M., Suh D.S., Kim C.D., Kim J.H., Birnbaum M.J., Bae S.S. (2008). Lysophosphatidic acid induces cell migration through the selective activation of akt1. Exp. Mol. Med..

[102] Li Y., Gonzalez M.I., Meinkoth J.L., Field J., Kazanietz M.G., Tennekoon G.I. (2003). Lysophosphatidic acid promotes survival and differentiation of rat schwann cells. J.Biol. Chem..

[103] Murga C., Fukuhara S., Gutkind J.S. (2000). A novel role for phosphatidylinositol 3-kinase beta in signaling from g protein-coupled receptors to akt. J. Biol. Chem..

[104] Baudhuin L.M., Cristina K.L., Lu J., Xu Y. (2002). Akt activation induced by lysophosphatidic acid and sphingosine-1-phosphate requires both mitogen-activated protein kinase kinase and p38 mitogen-activated protein kinase and is cell-line specific. Mol. Pharmacol..

[105] Kihara Y., Mizuno H., Chun J. (2015). Lysophospholipid receptors in drug discovery. Exp. Cell Res..

